# Fermi blockade of the strong electron–phonon interaction in modelled optimally doped high temperature superconductors

**DOI:** 10.1038/s41598-021-89059-w

**Published:** 2021-05-06

**Authors:** Andrey S. Mishchenko, Igor S. Tupitsyn, Naoto Nagaosa, Nikolay Prokof’ev

**Affiliations:** 1 RIKEN Center for Emergent Matter Science (CEMS), Wako, Saitama 351-0198 Japan; 2 Department of Physics, University of Massachusetts, Amherst, MA 01003 USA; 3 Department of Applied Physics, The University of Tokyo, 7-3-1 Hongo, Bunkyo-ku, Tokyo, 113-8656 Japan

**Keywords:** Materials science, Mathematics and computing, Physics

## Abstract

We study how manifestations of strong electron–phonon interaction depend on the carrier concentration by solving the two-dimensional Holstein model for the spin-polarized fermions using an approximation free bold-line diagrammatic Monte Carlo method. We show that the strong electron–phonon interaction, obviously present at very small Fermion concentration, is masked by the Fermi blockade effects and Migdal’s theorem to the extent that it manifests itself as moderate one at large carriers densities. Suppression of strong electron–phonon interaction fingerprints is in agreement with experimental observations in doped high temperature superconductors.

## Introduction

Discussions on the role of the electron–phonon interaction (EPI) in the physics of cuprate compounds with high superconducting transition temperatures (high $T_c$) have been going for decades^1–6^ without resulting in a consensus opinion. While the role of EPI in superconductivity is still under debate, its strong manifestations were clearly observed in numerous other phenomena in high $T_c$ materials^5–14^. The apparent puzzle is that strong EPI effects seen in spectroscopic data of undoped and weakly doped compounds become much less pronounced with hole doping^15–21^. Hence, having a clear picture of how the EPI effects change with the carrier concentration is of seminal importance for understanding the nature of unconventional superconductors where rigorous studies are hindered by the complexity of many-body fermion problem. Accurate results on the EPI in many-fermion systems may provide the way to reconcile the observed fingerprints of the strong EPI in the underdoped regime with successful descriptions of the strongly doped high $T_c$ materials by models based on direct electron-electron interactions alone.

More generally, it is a long standing fundamental problem to reveal how the Migdal’s theorem^22,23^ emerges at the large fermion concentration and eliminates the need for vertex corrections even for strong EPI, provided the Fermi-liquid state remains stable. Violations of the Migdal theorem in high temperature superconductors were discussed for decades^24^. Most studies^25,26^ only evaluate the lowest order corrections whereas our goal is to have a complete picture where all approximations, order-by-order, are compared to the exact answer as the fermion concentration approaches zero. The crossover between the two regimes is expected to take place at $\omega _{\text{ ph }} \sim \varepsilon _{F}$, where $\omega _{\text{ ph }}$ is the phonon frequency and $\varepsilon _{F}$ is the Fermi energy, and it can be addressed by the approximation free diagrammatic Monte Carlo methods^8,27–29^.

To this end, we consider a spin polarized (SP) two-dimensional (2D) lattice system in order to avoid system instabilities that would be triggered by the strong EPI in continuous and spin-balanced systems, such as structural transitions or a singlet on-site bipolaron formation at $\lambda \approx 0.5$ (in 2D)^30^ with the concomitant superconducting state. An essential feature of the SP Holstein model resembling that of the $t-J$ model near half-filling^31,32^ (which is prototypical for description of high $T_c$ superconductors) is that in both cases one can only create one hole per site. Strong repulsive electron-electron interactions obviously play a key role close to half-filling. However, our study is focused on the small density regime where Hubbard-type interactions can be treated perturbatively (after strong on-site repulsion is replaced with an exact solution of the two-body scattering problem) and result only in weak renormalization of electron properties. When the electron–phonon coupling is strong, repulsive electron-electron interactions are required for system stabilization against the bi-polaron instability, but otherwise their effects remain perturbative. This is precisely the situation that we are modeling at the microscopic level in our work.

## Exact numeric approach

In this work we employ the bold-line diagrammactic Monte Carlo (BDMC) technique developed for many-body systems with EPI in Ref.^29^. For the same system parameters the determinant Monte Carlo^33–35^ method would suffer from a severe sign problem. The dynamical mean-filed method (DMFT)^36,37^, would be inadequate because the EPI self-energy is strongly momentum dependent at low carrier concentration^29^, in violation of the key DMFT assumption. The BDMC technique is based on the expansion of irreducible free-energy Feynman diagrams in terms of exact electron, *G*, and bare, $D^{(0)}$, phonon propagators and is free from the above limitations. We consider phonon propagators $D^{(0)}$ as exact, fully renormalized/dressed functions as if they are already the result of an accurate ab-initio treatment^38,39^. Thus, we omit all diagrams that can be interpreted as the phonon proper self-energy insertion; accounting for phonon renormalization would have no significant effect on our results because we focus on the small carrier concentration limit. In more detail, see Ref.^29^, the electron self-energy $\Sigma ^{(m)}$ is expanded into the series of irreducible skeleton graphs up to the largest order *m* defined by the number of $D^{(0)}$ propagators, with self-consistency implemented by a feedback loop involving the solution of the algebraic Dyson equation, $[G(\mathbf {k},\omega _\ell )]^{-1} = [G^{(0)}(\mathbf {k},\omega _\ell )]^{-1} -$ $\Sigma ^{(m)}(\mathbf {k},\omega _\ell )$, in momentum, $\mathbf {k}$, and Matsubara frequency, $\omega _{\ell } = 2 \pi T(\ell +1/2)$, representation ($\ell$ is an integer).

## Model

The 2D Holstein model on a square lattice reads:

1 $$\begin{aligned} H=-t \!\! \sum _{<i,j>} c^{\dagger }_{i}c_{j}^{\,} + \omega _{\text{ ph }} \sum _{i}b_{i}^{\dagger } b_{i}^{\,} + g \sum _{i} c_{i}^{\dagger }c_{i}^{\,} \left( b_{i}^{\dagger } +b_{i}^{\,} \right) , \end{aligned}$$

where $c_{i}^{\dagger }$/$b_{i}^{\dagger }$ are standard notations for electron/phonon creation operators, *t* is the nearest neighbor hopping amplitude, $\omega _{\text{ ph }}=0.5t$ is the energy of the local optical mode, and *g* is the EPI coupling. The electron gas is spin-polarized and, hence, any site can be occupied by no more than one electron. It is standard to characterize the strength of the EPI by a dimensionless coupling constant $\lambda = g^2 / (4 \omega _{\text{ ph }} t)$. The lattice constant *a*, amplitude *t*, and Planck’s constant $\hbar$ are used to set units of length, energy, and time, respectively. In this study we chose $\lambda =1.07$ beyond the crossover from weak- to strong-coupling regimes for single polarons and the threshold for the singlet bipolaron formation. For convenient systematic error-free handling of the data in momentum space we perform simulations for finite systems with $16 \times 16$ sites, large enough to reproduce the infinite system results with high accuracy (see Supplementary Material). The temperature is set to $T=t/20$, which is an order of magnitude smaller than all energy scales of the model parameters. In the zero-density limit an alternative exact (numerically) diagrammatic Monte Carlo (DMC) approach for single polarons^27,28^ provides reference values for the ground state energy, $E_1=-4.891$, and the quasiparticle (QP) residue, $Z_1=0.238$.

## Results

**Figure 1:**
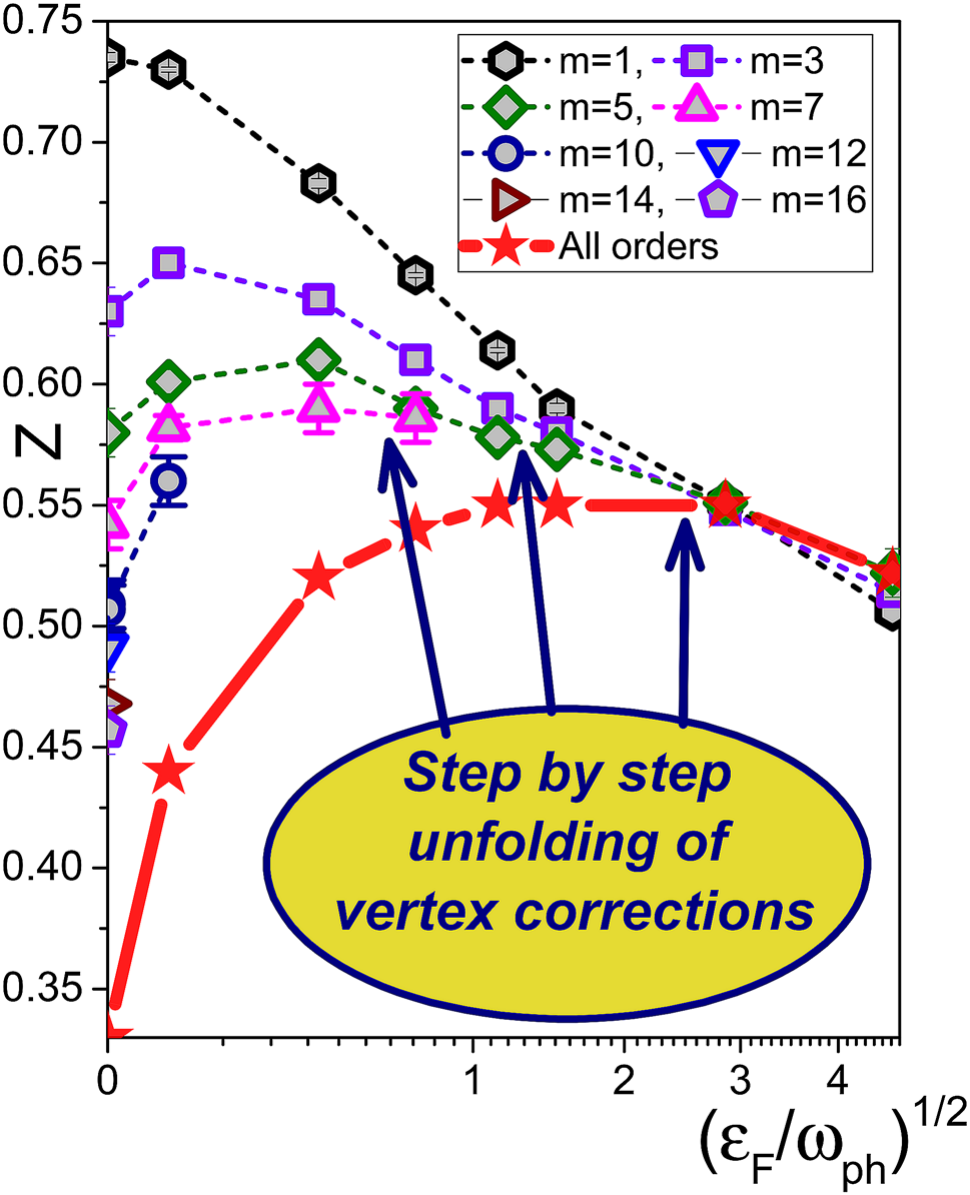
Quasi-particle residue at the Fermi (FS) as a function of ratio between the Fermi energy and phonon frequency without ($m=1$) and with vertex corrections ($m>1$). Symbols and dashed lines represent data obtained by skeleton expansions truncated at some finite order *m*. The solid red line with stars is obtained by extrapolation to the infinite diagram-order limit $m \rightarrow \infty$. The errorbars, if not visible, are smaller than the symbol sizes.

**Figure 2:**
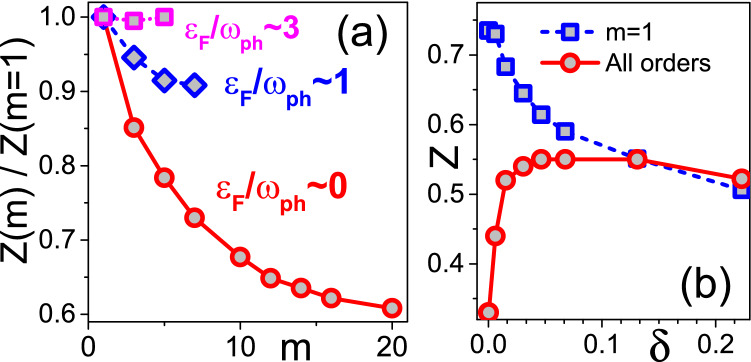
(**a**) Ratio between the quasi-particle residue deduced from diagrams up to order *m* and $m=1$ (neglecting vertex corrections). Circles, diamonds, and squares stand for $\gamma \rightarrow 0$ ($\delta =3.8 \times 10^{-4}$), $\gamma =0.71$ ($\delta =0.0308$), and $\gamma =2.86$ ($\delta =0.131$), respectively. (**b**) Quasi-particle residue at the Fermi (FS) as a function of carrier concentration $\delta$ (circles, infinite diagram-order limit) in comparison with the $m=1$ result (squares), see also Fig. 1.

**Figure 3:**
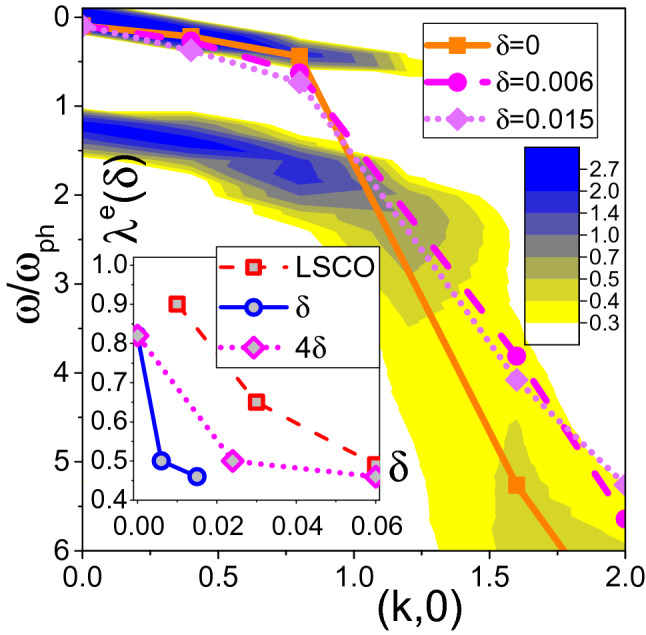
Contour plot of the spectral function intensity at $\delta =3.8 \times 10^{-4}$ with blue/yellow color used for the large/small intensity. Symbols connected with lines mark locations of the spectral density maxima, see also Fig. 4, for: $\delta =3.8 \times 10^{-4}$ (squares connected by the solid line), $\delta \approx 0.006$ (circles connected by the dashed line), and $\delta \approx 0.015$ (diamonds connected by the dotted line). In the inset we present the effective coupling constant $\lambda ^e$ deduced from the scaling relation (2) using experimental data for LSCO^21^ (squares connected by a dashed line) and locations of theoretical spectral density maxima in Fig. 4 (circles connected by a solid line). We also re-plot the same theoretical data by using $4 \delta$ for the horizontal axis (diamonds connected by a dotted line). Spectral densities were computed for self-energies evaluated up to order $m=16$ ($\delta =3.8 \times 10^{-4}$), $m=7$ ($\delta = 0.006$), and $m=5$ ($\delta = 0.015$). These expansion orders are enough to have converged results for the corresponding carrier density (see Supplementary Material, Table I). Prepared by OriginPro 2016, http://OriginLab.com.

Our main results are presented in Figs. 1, 2, and 3. Figure 1 shows the dependence of the QP residue on the adiabaticity ratio $\gamma = \varepsilon _{F} / \omega _{\text{ ph }}$. One can see in Fig. 1 that at large $\gamma \ge 3$ the Migdal’s theorem ensures that vertex corrections are small and the lowest-order $m=1$ skeleton diagram for self-energy (also known, depending on the context, as the non-crossing, the self-consistent Born, and the Eliashberg approximations) well describes the EPI renormalization even at strong coupling. In contrast, for smaller values of $\gamma$ one has to account for high-order vertex corrections; up to order $m=7$ at $\gamma =1$ and all the way to $m>20$ for $\gamma \rightarrow 0$ with extrapolation to the infinite diagram-order limit. An immediate conclusion is that EPI strongly suppresses the QP residue to values smaller that 0.5 (indicative of strong coupling) only at a rather small filling factor when $\gamma < 1$

.

In Fig. 2a we further quantify the role of vertex corrections at low and high carrier density (or occupation number per site), $\delta$, in both adiabatic and anti-adiabatic regimes. Vertex corrections become important at $\gamma <3$, and at low values of $\gamma$ and $\delta$ it is not sufficient to take into account just $m=2$, or even $m=3$ contributions; in this parameter regime the convergence is reached only for $m \ge 16$ in the skeleton expansion, see Fig. 2a). Figure 2b is complementary to Fig. 1 by presenting the data as a function of the carrier concentration $\delta$ instead of $\gamma$. Signatures of strong EPI are observed only at $\delta < 0.1$ that roughly corresponds to $\gamma \approx 1$, which can be interpreted as the “Fermi blockade” of EPI manifestations at large concentration by the Pauli exclusion principle.

The key conclusion that clear signatures of strong EPI are limited to small doping is consistent with experimental findings for high $T_c$ superconductors^15,19–21^. It is not restricted to specific model features or choice of parameters. For example, smaller values of the phonon frequency lead to sharper crossover between the weak and strong coupling regimes for single Holstein polarons (see Supplementary Material). Therefore, the crossover presented in Figs. 1 and 2 will be more pronounced for smaller phonon frequencies. Besides, similar results are obtained for the spin-balanced case when the bi-polaron instability is prevented by adding an on-site Hubbard repulsion, $H_{\text{ Hub }} = \sum _{i} U n_{i\uparrow } n_{i\downarrow }$ with $n_{i\sigma } = c_{i\sigma }^{\dagger }c_{i\sigma }^{\,}$, to the Hamiltonian (see Supplementary Material) (the corresponding simulations are far more demanding).

**Figure 4:**
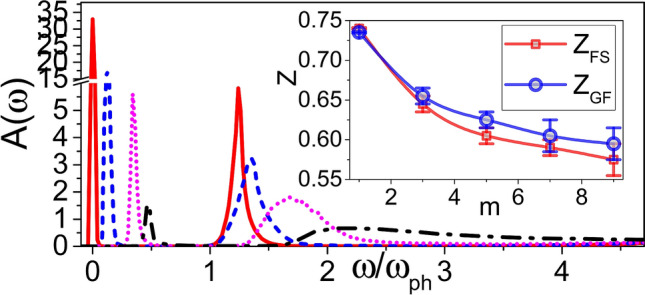
Spectral functions $A(\omega )$ at different momenta for $\delta =3.8 \times 10^{-4}$ from $m=16$ simulations: $q=(0,0)$ (red solid line), $q=(\pi /8,0)$ (blue dashed line), $q=(2\pi /8,0)$ (magenta dotted line), and $q=(3\pi /8,0)$ (black dash-dotted line). Energy zero was set at the value of the QP dispersion relation at $q=0$. Inset: Order-by-order comparison between the two alternative procedures for computing the quasi-particle residue at $q=0$: (i) using standard Fermi liquid relations at the Fermi surface, $Z_{FS}$, and (ii) from the lowest-frequency peak in the spectral function, $Z_{GF}$.

One evidence for Fermi blockade of the EPI with doping comes from angle resolved photoemission experiments^15^. It was shown that the ratio $v_{\text{ high }}/v_{\text{ low }}$ between the phase velocities of the dispersion relation above and below the Debye frequency, decreases with doping. Our simulations reveal a similar trend, see Fig. 3. The QP dispersion relation $\omega (\mathbf { k})$ was obtained from the energy of the lowest peak in the Lehmann spectral function, see Fig. 4, extracted from the imaginary time Matsubara Green function $G(\tau )$ by the stochastic optimization with consistent constraints method of analytic continuation^28,40^.

Calculations of the frequency dependent optical conductivity^19^ and angle resolved photoemission spectra^21^ in the low-concentration limit (one hole) of the $t-J$-Holstein model revealed that the experimental dependence of both quantities on $\delta$ can be reproduced theoretically if one introduces effective EPI coupling constant $\lambda ^e(\delta )$ that decreases with doping. It can be deduced from the photoemission spectra using scaling relation^21^

2 $$\begin{aligned} \lambda ^{e} = \sqrt{ \frac{v_{\text{ high }}-v_{\text{ low }}}{20v_{\text{ low }} } \; , } \end{aligned}$$

derived from nonperturbative calculations for the $t-J$-Holstein model, where $v_{\text{ low }}$ ($v_{\text{ high }}$) is the velocity above (below) the kink energy $\omega _{\text{ ph }}$.

Kinks in the photoemission spectra are experimentally observed on the side of the Fermi surface where the electron (or hole) bandwidth exceeds the phonon frequency. Additional spectral features (rather typical in the small density limit when the Fermi energy is smaller than $\omega _{ \text{ ph }}$) are associated with the phonon sidebands^41^. Approximation free studies of the kink phenomenon show^21^ that, in general, the kink coexists with the phonon sidebands and only poor resolution or additional damping mechanisms smear out the sidebands^21,42^ leading to a prototypical picture of a sudden velocity change. Note, at least one phonon sideband, seen as the doubling of the spectral peak around the kink energy, is present in most theoretical calculations^7,21,42,43^ and this is precisely what we see in Fig. 3 at $\omega / \omega _{ \text{ ph }} \approx 1$ and small momentum $k<1$ .

We compare $\lambda ^e(\delta )$ deduced from experimental data of Ref.^21^ with our theoretical analysis in the inset of Fig. 3, dashed versus solid line. To have a meaningful quantitative comparison we also need to account for the difference between the non-degenerate spectrum of the spin-polarized Holstein model and fourfold degenerate ground state minimum of the experimental system. To this end we re-plot theoretical data using $4\delta$ for the carrier concentration (dotted line). We observe semi-quantitative agreement between the theory and experiment despite a number of significant differences between the real systems and SP Holstein model at the microscopic level.

## Discussion

The violation of Migdal’s theorem for $T=0$ is apparent in Fig. 1 for all filling factors except the two largest ones. At the lowest carrier concentrations the condition $\varepsilon _{F} \gg T$ does not hold any more, but this fact is barely relevant for the discussion because the theorem is severely violated well before that, at $\varepsilon _{F} \sim \omega _{\text{ ph }} \gg T$. Thus, our finite temperature results are still valid for interpretation of the EPI suppression in high $T_c$ materials, which is observed from low to room temperatures^15,19–21^.

The suppression of the EPI effects considered here is not related to the weakening of the EPI due to screening at larger doping, see e.g.^44,45^, because we do not change the coupling strength $\lambda$ and the phonon propagator with doping. In addition to the discussed EPI mechanism of how electronic properties are renormalized in cuprates, one has to consider electron-electron interactions and the emergent strong spin-fluctuations, see, e.g. discussions in Refs.^46,47^. However, these additional important considerations are most relevant at large electron concentration close to half-filling, while the doping dependence discussed in this work takes place in the opposite limit.

## Conclusion

We obtained approximation-free results for the concentration dependence of the quasiparticle residue *Z* and kink caused by the strong electron–phonon interaction in the spin-polarized two-dimensional Holstein model on the square lattice. We demonstrated that clear signatures of strong electron–phonon coupling at small carrier concentration are quickly suppressed for Fermi energies exceeding the phonon frequency. Our results provide detailed account for importance of high-order vertex corrections across the adiabatic crossover and demonstrate that Fermi blockade of the electron–phonon interaction and irrelevance of vertex corrections both proceed in agreement with the Migdal’s theorem. This picture explains experimental results reporting radical weakening of the electron–phonon coupling effects in lightly doped high temperature superconductors. The Fermi blockade phenomenon is not restricted to high temperature superconductors and has to be observed in any material showing strong electron–phonon effects at small electron concentration.

## Methods

All data for the QP residues at the FS, also denoted as $Z_{FS}$, were deduced from the Fermi-liquid relation, $Z_{FS} =[1+d]^{-1}$, with $d = \partial Re[\Sigma (k_F,\omega )]/\partial \omega \vert _{\omega =0}$. In the low-temperature limit, the self-energy derivative at zero frequency is accurately obtained from the ratio $-\text{ Im }[\Sigma (k_F,\ell )]/\omega _\ell$ at the lowest Matsubara frequencies. As expected, this procedure works perfectly at large carrier concentration. However, in the zero density limit the Fermi surface shrinks to a point at zero momentum, and the entire protocol becomes questionable. Spectral density offers an alternative way of computing the QP residue from the integrated weight of the lowest frequency peak (we denote it as $Z_{GF}$), see Fig. 4. Somewhat surprisingly, we find that even in the zero-density limit both procedures produce consistent results at any expansion order *m*, see inset in Fig. 4. At small, but finite concentration $\delta =0.01526$ (or $\gamma =0.334$), with Fermi-momentum $k_F \approx \pi /8$ the agreement is even more precise: at order $m=5$ we find that $Z_{FS}=0.605$ and $Z_{GF}(k_F=\pi /8)=0.611$.

**Figure 5:**
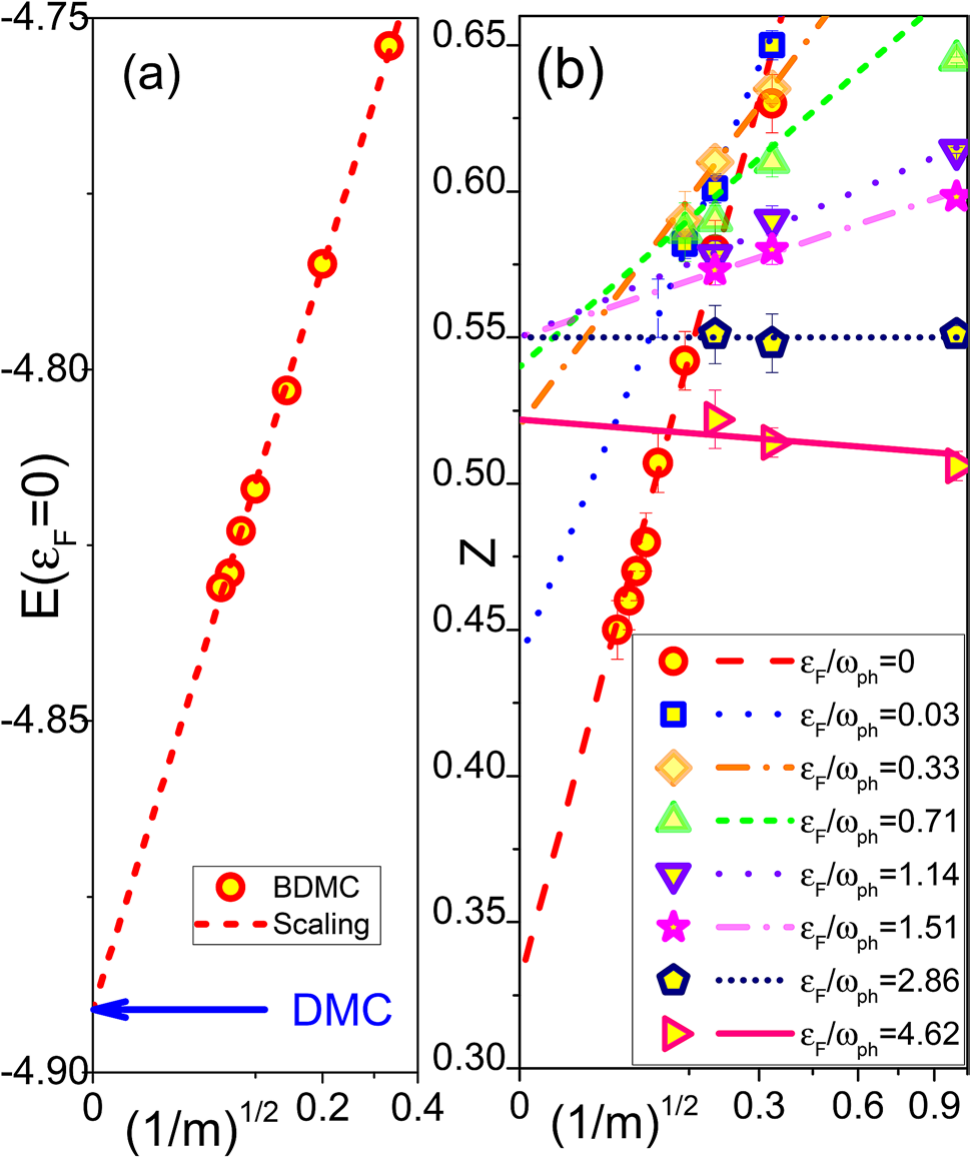
Finite expansion-order corrections to the polaron energy (**a**) and QP residue (**b**) revealing linear scaling with $m^{-1/2}$. (**a**) BDMC data (circles) and the scaling law $a+b/\sqrt{m}$ (dashed line) for the ground state energy at $\delta =3.8 \times 10^{-4}$. The DMC result at $\delta =0$ is shown by the blue arrow. (**b**) BDMC data (symbols) and the scaling laws $a+b/\sqrt{m}$ (lines) for the quasi-particle residue.

As already mentioned in connection with Figs. 1 and 2a, at small doping the skeleton expansion needs to go beyond $m=16$ in order to obtain correct results for the QP residue. However, both *Z* and the polaron energy *E* at the FS accurately follow an empirical scaling relation, $a + b/ \sqrt{m}$, at any carrier concentration $\delta$, see Fig. 5. This allows us to perform an extrapolation to the infinite-order limit to eliminate the remaining systematic error as shown in Figs. 1, 2. The extrapolation procedure is validated by an excellent agreement between the BDMC result for the ground state energy of single-polarons, $E(m \rightarrow \infty ) =-4.89$ and the DMC benchmark $E_1=-4.891$. The single polaron zero temperature residue $Z_1=0.238$ is renormalized to $Z_1(\beta =20) \approx 0.31$ due to finite temperature projection of the low energy self-trapping states^48,49^ (see Supplementary Material) which is also consistent with extrapolated value $Z(m \rightarrow \infty ) \approx 0.33$.

## Supplementary Information

Supplementary Information.

## Data Availability

*Accession codes* The codes for BDMC are available at GitHub webpage https://github.com/andry-mi-63/BDMC-1 and the codes for analytic continuation are available at JST ImPACT project webpage https://www.jst.go.jp/impact/hp_yamamoto/en/index.html in "Quantum Simulation Open program". Data presented in figures are available from the corresponding author on reasonable request.

## References

[1] Anderson PW (1997). The Theory of Superconductivity in the High-T_c Cuprate Superconductors.

[2] Alexandrov AS (1996). Bipolaron anisotropic flat bands, hall mobility edge, and metal-semiconductor duality of overdoped high-$${T}_{c}$$ oxides. Phys. Rev. B.

[3] Anderson PW (2007). Is there glue in cuprate superconductors?. Science.

[4] Alexandrov AS (2007). Bose-Einstein condensation of strongly correlated electrons and phonons in cuprate superconductors. J. Phys. Condens. Matter.

[5] Gunnarsson O, Rösch O (2008). Interplay between electron-phonon and coulomb interactions in cuprates. J. Phys. Condens. Matter.

[6] Mishchenko AS (2009). Electron-phonon coupling in underdoped high-temperature superconductors. Physics-Uspekhi.

[7] Rösch O, Gunnarsson O (2004). Electron-phonon interaction in the $$t{-}J$$ model. Phys. Rev. Lett..

[8] Mishchenko AS, Nagaosa N (2004). Electron-phonon coupling and a polaron in the $$t{-}J$$ model: From the weak to the strong coupling regime. Phys. Rev. Lett..

[9] Rösch O (2005). Polaronic behavior of undoped high-$${T}_{c}$$ cuprate superconductors from angle-resolved photoemission spectra. Phys. Rev. Lett..

[10] Cataudella V, De Filippis G, Mishchenko AS, Nagaosa N (2007). Temperature dependence of the angle resolved photoemission spectra in the undoped cuprates: Self-consistent approach to the $$t{-}J$$ Holstein model. Phys. Rev. Lett..

[11] De Filippis G, Cataudella V, Mishchenko AS, Nagaosa N (2007). Nonlocal composite spin-lattice polarons in high temperature superconductors. Phys. Rev. Lett..

[12] De Filippis G (2012). Quantum dynamics of the Hubbard-Holstein model in equilibrium and nonequilibrium: Application to pump-probe phenomena. Phys. Rev. Lett..

[13] Novelli F (2014). Witnessing the formation and relaxation of dressed quasi-particles in a strongly correlated electron system. Nat. Commun..

[14] Farina D (2018). Electron-phonon coupling in the undoped cuprate $${{\rm YBa}_{2}{\rm Cu}}_{3}{\rm O}_{6}$$ estimated from Raman and optical conductivity spectra. Phys. Rev. B.

[15] Lanzara A (2001). Evidence for ubiquitous strong electron-phonon coupling in high-temperature superconductors. Nature.

[16] Johnson PD (2001). Doping and temperature dependence of the mass enhancement observed in the cuprate $${{\rm Bi}_{2}{\rm Sr}}_{2}{\rm CaCu}_{2}{O}_{8+\delta }$$. Phys. Rev. Lett..

[17] Shen KM (2004). Missing quasiparticles and the chemical potential puzzle in the doping evolution of the cuprate superconductors. Phys. Rev. Lett..

[18] Zhou XJ (2005). Multiple bosonic mode coupling in the electron self-energy of $$({{\rm La}_{2-x}{\rm Sr}}_{x}){\rm CuO}_{4}$$. Phys. Rev. Lett..

[19] Mishchenko AS (2008). Charge dynamics of doped holes in high $${T}_{c}$$ cuprate superconductors: A clue from optical conductivity. Phys. Rev. Lett..

[20] Carbone F, Yang D-S, Giannini E, Zewail AH (2008). Direct role of structural dynamics in electron-lattice coupling of superconducting cuprates. Proc. Natl. Acad. Sci..

[21] Mishchenko AS (2011). Polaronic metal in lightly doped high-t$$T_c$$ cuprates. EPL.

[22] Migdal AB (1958). Interaction between electrons and lattice vibrations in a normal metal. Zh. Eksperim. i Teor. Fiz..

[23] Husanu M-A (2020). Electron-polaron dichotomy of charge carriers in perovskite oxides. Commun. Phys..

[24] Ummarino GA, Gonnelli RS (1997). Breakdown of Migdal’s theorem and intensity of electron-phonon coupling in high-$${T}_{c}$$ superconductors. Phys. Rev. B.

[25] Grimaldi C, Pietronero L, Strässler S (1995). Nonadiabatic superconductivity: Electron-phonon interaction beyond Migdal’s theorem. Phys. Rev. Lett..

[26] Schrodi F, Oppeneer PM, Aperis A (2020). Full-bandwidth Eliashberg theory of superconductivity beyond Migdal’s approximation. Phys. Rev. B.

[27] Prokof’ev NV, Svistunov BV (1998). Polaron problem by diagrammatic quantum Monte Carlo. Phys. Rev. Lett..

[28] Mishchenko AS, Prokof’ev NV, Sakamoto A, Svistunov BV (2000). Diagrammatic quantum Monte Carlo study of the fröhlich polaron. Phys. Rev. B.

[29] Mishchenko AS, Nagaosa N, Prokof’ev N (2014). Diagrammatic Monte Carlo method for many-polaron problems. Phys. Rev. Lett..

[30] Macridin A, Sawatzky GA, Jarrell M (2004). Two-dimensional Hubbard-Holstein bipolaron. Phys. Rev. B.

[31] Kane CL, Lee PA, Read N (1989). Motion of a single hole in a quantum antiferromagnet. Phys. Rev. B.

[32] Dagotto E (1994). Correlated electrons in high-temperature superconductors. Rev. Mod. Phys..

[33] Blankenbecler R, Scalapino DJ, Sugar RL (1981). Monte Carlo calculations of coupled boson-fermion systems. I. Phys. Rev. D.

[34] White SR (1989). Numerical study of the two-dimensional Hubbard model. Phys. Rev. B.

[35] Noack RM, Scalapino DJ (1993). Green’s-function self-energies in the two-dimensional Holstein model. Phys. Rev. B.

[36] Georges A, Kotliar G, Krauth W, Rozenberg MJ (1996). Dynamical mean-field theory of strongly correlated fermion systems and the limit of infinite dimensions. Rev. Mod. Phys..

[37] Bauer J, Han JE, Gunnarsson O (2011). Quantitative reliability study of the Migdal-Eliashberg theory for strong electron-phonon coupling in superconductors. Phys. Rev. B.

[38] Brovnman EG, Kagan Y (1967). The phonon spectrum of metals. Sov. Phys. JETP.

[39] Tupitsyn IS, Mishchenko AS, Nagaosa N, Prokof’ev N (2016). Coulomb and electron-phonon interactions in metals. Phys. Rev. B.

[40] Goulko O, Mishchenko AS, Pollet L, Prokof’ev N, Svistunov B (2017). Numerical analytic continuation: Answers to well-posed questions. Phys. Rev. B.

[41] Krsnik J (2020). Manifestations of the electron-phonon interaction range in angle-resolved photoemission spectra. Phys. Rev. B.

[42] Devereaux TP, Cuk T, Shen Z-X, Nagaosa N (2004). Anisotropic electron-phonon interaction in the cuprates. Phys. Rev. Lett..

[43] Veenstra CN, Goodvin GL, Berciu M, Damascelli A (2010). Elusive electron-phonon coupling in quantitative analyses of the spectral function. Phys. Rev. B.

[44] Sherman EY, Ambrosch-Draxl C (2000). Multiband electron-phonon coupling in the cuprates: Raman scattering and charge fluctuations. Phys. Rev. B.

[45] Johnston S (2010). Systematic study of electron-phonon coupling to oxygen modes across the cuprates. Phys. Rev. B.

[46] Dahm T (2009). Strength of the spin-fluctuation-mediated pairing interaction in a high-temperature superconductor. Nat. Phys..

[47] Anzai H (2017). A new landscape of multiple dispersion kinks in a high-tc cuprate superconductor. Sci. Rep..

[48] Bonča J, Trugman SA, Batistič I (1999). Holstein polaron. Phys. Rev. B.

[49] Mishchenko AS, Nagaosa N, Prokof’ev NV, Sakamoto A, Svistunov BV (2002). Self-trapping of polarons in the Rashba-Pekar model. Phys. Rev. B.

