# Supplementary material for: Fermi blockade of the strong electron–phonon interaction in modelled optimally doped high temperature superconductors
Source: Sci Rep. 2021 May 6;11:9699. doi: 10.1038/s41598-021-89059-w (PMC8102534; doi:10.1038/s41598-021-89059-w)
Supplement: Supplementary file 1 — Supplementary Information. [file 41598_2021_89059_MOESM1_ESM.pdf]

Supplementary material for ”Fermi blockade of the strong electron-phonon interaction in modelled optimally doped high temperature superconductors"

Andrey S. Mishchenko[1,*], **Igor S. Tupitsyn**[2], **Naoto Nagaosa**[1,3], **and Nikolay Prokof'ev**[2]

[1]RIKEN Center for Emergent Matter Science (CEMS), Wako, Saitama 351-0198, Japan
[2]Department of Physics, University of Massachusetts, Amherst, MA 01003, USA
[3]Department of Applied Physics, The University of Tokyo 7-3-1 Hongo, Bunkyo-ku, Tokyo 113-8656, Japan
[*]mishchenko@riken.jp

Here we provide additional details on calculations performed for the 2D Holstein model described in the main text. The lattice constant a, hopping amplitude t, and Planck's constant $\hbar$ are used to set units of length, energy, and time, respectively. The phonon frequency $\omega_{\text{ph}} = 0.5t$ is nearly an order of magnitude smaller than the particle bandwidth, and the dimensionless coupling constant $\lambda = 1.07$ corresponds to the strong coupling regime (see also below).

1 Size dependence

To check whether the system size $N \times N = 16 \times 16$ is sufficient to reproduce properties of the Holstein model for single polarons when the largest finite-size effects are expected, we calculated various characteristics of the polaron by the diagrammatic Monte Carlo[1,2] and compared them with known infinite lattice results. In the simulations of finite lattice all momenta in the reciprocal space also form a lattice

$$k_{x,y} = (2\pi/N)j, \;\; -N/2 \leq j < N/2 .$$

In Fig. 1 we show how the polaron energy, E, and quasiparticle residue, Z, depend on the lattice size for $N = 4, 8, 16, 32, 64, 128, \infty$, and conclude that $N = 16$ results reproduce the infinite system limit with accuracy of three to four significant digits.

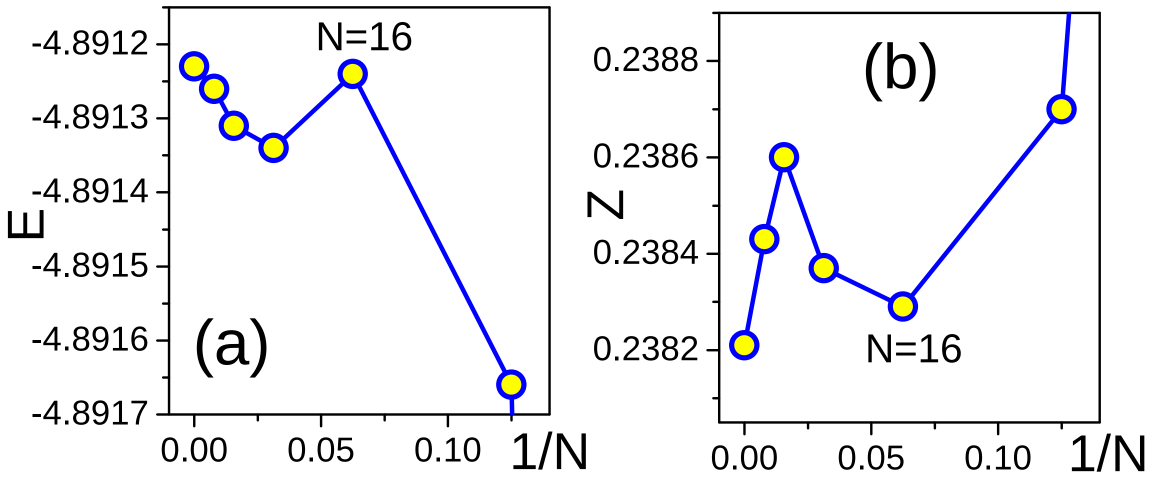

Figure 1. System size dependence of the polaron energy (a) and quasiparticle residue (b).

2 Convergence of the BLDMC series as a function of carrier density

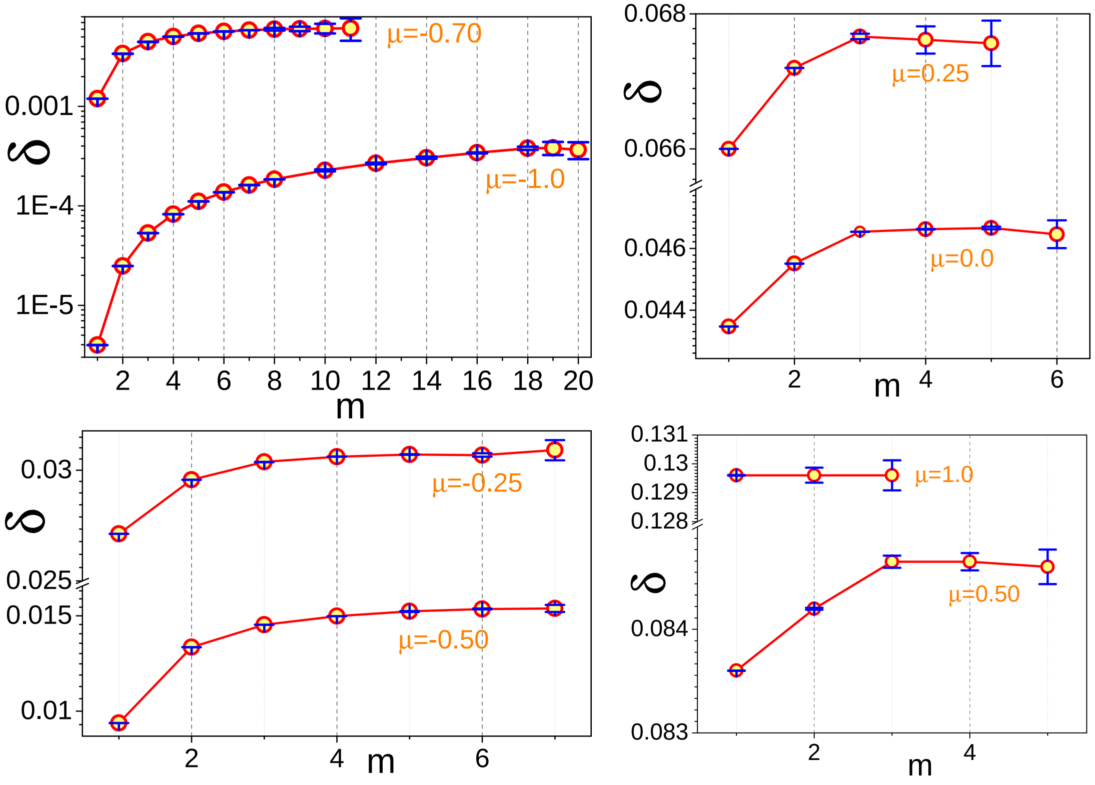

Figure 2. Fermion density dependence on the order of the self-consistent skeleton expansion m for different values of the initial chemical potential μ.

μ	δ	e_F/ω_{ph}	Convergent m
1.0	0.131	2.86	1
0.5	0.085	2.0	3
0.25	0.068	1.51	4
0.0	0.047	1.14	4
-0.25	0.031	0.71	4
-0.5	0.015	0.33	5
-0.7	0.006	0.028	7
-1.0	3.8×10^{-4}	0	16

Table 1. Relations between the chemical potential, μ, fermion density per site, δ, and ratio between the Fermi energy and phonon frequency e_F/ω_{ph}. To establish them one needs to account for skeleton diagrams up to order m.

Convergence properties of the skeleton expansion strongly depend on the fermion density δ (or chemical potential, μ, in the grand canonical ensemble). In Fig. 2. we present our BLDMC data for density dependence on the expansion order at low temperature $T = t/20$ and different values of μ. At low density one needs to account for vertex corrections up to order 16 to obtain converged results. Note that the chemical potential μ is not directly related to the Fermi energy counted counted from the bottom of the dispersion relation which is strongly renormalized by interactions. Table 1 provides final relations between all quantities, including the required expansion order.

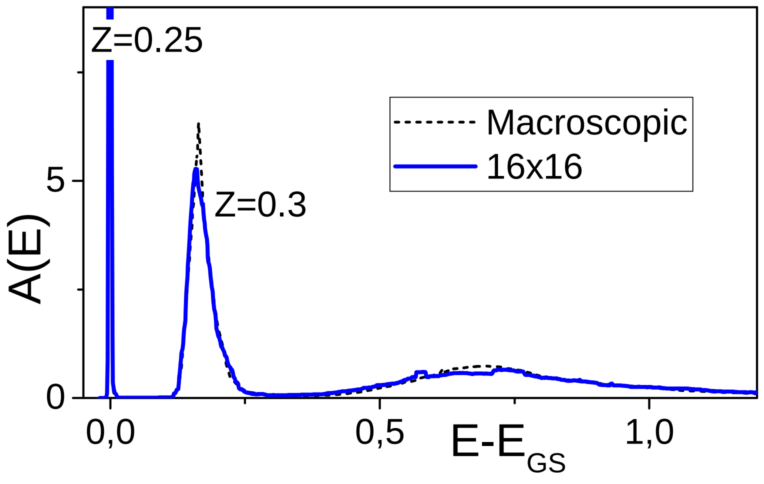

Figure 3. Spectral function of a single polaron in the infinite (black dashed line) and finite 16x16 (solid blue line) systems.

3 Ground state energy, Z-factor, and spectral function of a single polaron

In Fig. 3 we present the spectral function of a single polaron in the infinite in finite $N^2 = 16^2$ systems. Nearly perfect agreement (well within the analytic continuation procedure uncertainties) proves that finite-size effects in this case are negligible not only for ground state energies but also for excited states.

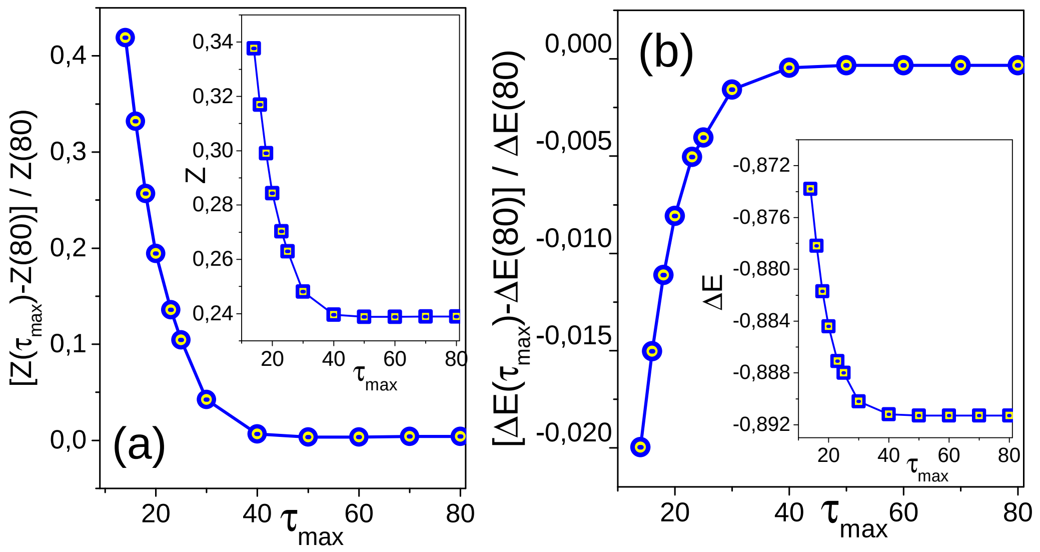

Figure 4. Saturation of estimates for the quasiparticle residue (a) and ground state energy (b) as a function of τ_{max}. For each value of τ_{max} the imaginary-time Green's function on the $[0.95\tau_{max}, 1.05\tau_{max}]$ interval was fitted by a simple exponential function to extract Z and ΔE, see text.

To determine the quasiparticle residue and interaction induced energy shift, $\Delta E = E - (-4t)$, we rely on the standard reliable method: at large imaginary time the asymptotic decay of the Green's function is given by

$$G(\tau) \to_{\tau\to\infty} Z \exp(-\Delta E \tau) ,$$

see[1,2], allowing one to extract Z and ΔE from a simple exponential fit. The leading correction decays with exponent controlled by the lowest excited state (the second polaron state according to the spectral density analysis). In Fig. 4 we show how Z and E

estimates change when we move the fitting interval $[0.95\tau_{max}, 1.05\tau_{max}]$ to larger values of τ_{max}. It is clear from Fig. 3 that the energy dependence on τ_{max} within the range $\tau_{max} \in [14, 80]$ is very weak (about 2%). This is in sharp contrast, with the quasiparticle residue estimates: Z increases by nearly 40% when τ_{max} decreases from $\tau_{max} = 80$ to $\tau_{max} = 14$. This sensitivity explains the discrepancy between the calculations performed at finite temperature $T = t/20$ and at $T = 0$. We attribute it to the presence of the second polaron state with comparable Z factor and relatively small excitation energy $E_2 - E_G \approx 0.17t$.

4 Relation of the single polaron parameters and results of extrapolation procedure for BDMC data

The extrapolation procedure is validated by an excellent agreement between the BDMC result for the ground state energy of single-polarons, $E(m \to \infty) = -4.89$ and the DMC benchmark $E_1 = -4.891$. In the same limit, the extrapolated result for the QP residue $Z(m \to \infty) \approx 0.33$ turns out to be larger than that for single polarons, $Z_1 = 0.238$. The reason for the discrepancy is a combination of the finite temperature effect and self-trapping phenomenon[3,4], manifesting itself as a second, low energy, $E_2 - E_1 \approx 0.17 < \omega_{\text{ph}}$, excited polaron state with rather large spectral weight, $Z_2 \approx 0.3$, clearly observed in the spectrum of single polarons at $T = 0$, see Fig. 3. Because of this soft excitation, the standard procedure of extracting Z from the large-τ asymptotic behavior of the imaginary time Green function $G(\tau)$[1,2] turns out to be sensitive to the choice of the large imaginary time used to fit the data (for $\tau < 40$), whereas the estimate for energy remains accurate even for $\tau < 20$, see Fig. 4 . Therefore, at $T = t/20$ we detect the QP weight that overestimates Z_1 of single polarons in the ground state. Semi-quantitatively, the finite temperature BDMC result can be understood from the relation $Z_{\beta/2} = Z_1 + Z_2 \exp[-(\beta/2)(E_2 - E_1)] \approx 0.31$, which accounts for the activated second polaron state contribution at $\tau_{max} = \beta/2$.

5 Dependence of the crossover to the strong coupling regime on the phonon frequency

We studied how the ground state energy E, quasiparticle-residue Z, and average number of phonons N_{ph} in the polaron cloud, for a single Holstein polaron depend on the dimesionless coupling constant λ and the phonon frequency, see Fig. 5. One can see that the crossover between the weak and strong coupling regimes is more sharp for smaller values of the phonon frequency. Besides, it occurs at smaller values of λ for smaller values of ω_{ph}.

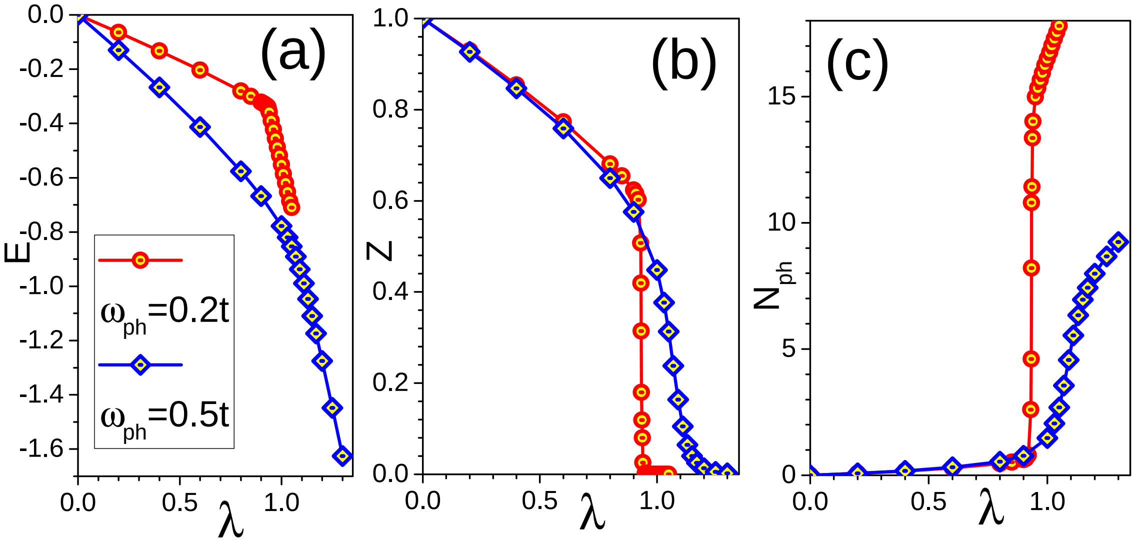

Figure 5. Dependence of (a) ground state energy E, (b) quasiparticle-residue Z, and (c) average number of phonons N_{ph} in the polaron cloud on the coupling constant λ for phonon frequencies $\omega_{\text{ph}} = 0.5t$ (blue dimonds) and $\omega_{\text{ph}} = 0.2t$ (red circles).

6 Filling factor dependence of the quasiparticle resudue Z in the Hubbard-Holstein model

The Hubbard-Holstein Hamiltonian on a square lattice reads:

$$H = -t \sum_{<i,j,s>} c_{is}^{\dagger} c_{js} + \omega_{\text{ph}} \sum_i b_i^{\dagger} b_i + g \sum_{is} c_{is}^{\dagger} c_{is} \left(b_i^{\dagger} + b_i \right) + \sum_i U n_{i\uparrow} n_{i\downarrow}, \quad (1)$$

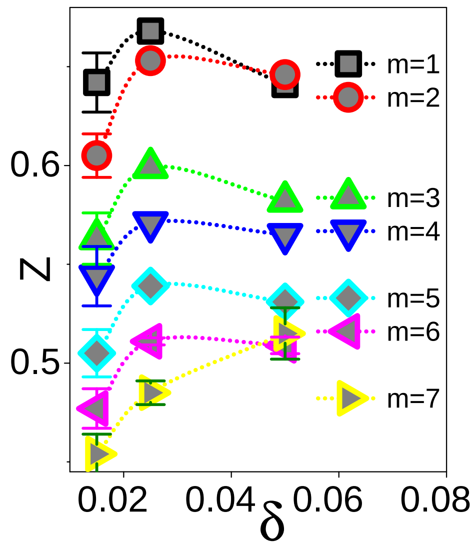

Figure 6. Quasi-particle residue at the Fermi (FS) as a function of small carrier concentration δ for Hubbard-Holstein model with $U = 2t$ and $\lambda = 1.07$ within the diagrammatic expansion truncated at different maximal order $1 \leq m \leq 7$.

where $c^{\dagger}_{is}/b^{\dagger}_{i}$ are standard notations for electron/phonon creation operators on site i with spin $s = \uparrow / \downarrow$, t is the nearest neighbor hopping amplitude, $n_{is} = c^{\dagger}_{is} c_{is}$, $\omega_{\text{ph}} = 0.5t$ is the energy of the local optical mode. U is the repulsive on-site repulsion, and g is the EPI coupling. The dimensionless EPI coupling constant $\lambda = g^2/(4\omega_{\text{ph}} t)$ is chosen to be $\lambda = 1.07$, i.e. the same as in the main text. We find that the bi-polaron instability is prevented when the on-site repulsion is increased to $U = 2t$. Concentration dependence of the quasiparticle residue at the Fermi surface for this model is presented in Fig. 6. The DiagMC simulation for model (1) is far more demanding compared to the spin-polarized case discussed in the main text, and without additional algorithmic developments cannot be carried out to expansion orders higher than ($m = 7$).

References

1. Prokof'ev, N. V. & Svistunov, B. V. Polaron Problem by Diagrammatic Quantum Monte Carlo. *Phys. Rev. Lett.* **81**, 2514-2517 (1998).

2. Mishchenko, A. S., Prokof'ev, N. V., Sakamoto, A. & Svistunov, B. V. Diagrammatic quantum Monte Carlo study of the Fröhlich polaron. *Phys. Rev. B* **62**, 6317-6336 (2000).

3. Bonča, J. and Trugman, S. A. & Batistić I. Holstein polaron. *Phys. Rev. B* **60**, 1633-1642 (1999).

4. Mishchenko, A. S., Nagaosa, N., Prokof'ev, N. V., Sakamoto, A. & Svistunov, B. V. Self-trapping of polarons in the Rashba-Pekar model. *Phys. Rev. B* **66**, 020301 (2002).
